# Rapid Multiplexed Detection on Lateral-Flow Devices Using a Laser Direct-Write Technique

**DOI:** 10.3390/bios8040097

**Published:** 2018-10-20

**Authors:** Peijun J. W. He, Ioannis N. Katis, Robert W. Eason, Collin L. Sones

**Affiliations:** Optoelectronics Research Centre, University of Southampton, Southampton SO17 1BJ, UK; I.Katis@soton.ac.uk (I.N.K.); rwe@orc.soton.ac.uk (R.W.E.); cls@orc.soton.ac.uk (C.L.S.)

**Keywords:** lateral-flow device, multiplexed detection, laser direct-write, biosensors, inflammation detection

## Abstract

Paper-based lateral flow devices (LFDs) are regarded as ideal low-cost diagnostic solutions for point-of-care (POC) scenarios that allow rapid detection of a single analyte within a fluidic sample, and have been in common use for a decade. In recent years, there has been an increasing need for rapid and simultaneous detection of multiple analytes present within a single sample and to facilitate this, we report here a novel solution—detection using a multi-path LFD created via the precise partitioning of the single flow-path of a standard LFD using our previously reported laser direct-write (LDW) technique. The multiple flow-paths allow the simultaneous detection of the different analytes individually within each of the parallel channels without any cross-reactivity. The appearance of coloured test lines in individual channels indicates the presence of the different analytes within a sample. We successfully present the use of a LDW-patterned multi-path LFD for multiplexed detection of a biomarker panel comprising C-reactive protein (CRP) and Serum amyloid A-1 (SAA1), used for the diagnosis of bacterial infections. Overall, we demonstrate the use of our LDW technique in the creation of a novel LFD that enables multiplexed detection of two inflammation markers within a single LFD providing a detection protocol that is comparatively more efficient than the standard sequential multiplexing procedure.

## 1. Introduction

Across a wide range of fields that include not just clinical diagnostics but also areas such as food safety testing and environmental assessment etc., the need for simple point-of-care (POC) testing solutions has become increasingly evident in recent years [1,2,3]. The World Health Organisation (WHO) has also defined a set of essential requirements to which POC diagnostic tools/tests, developed for the challenging needs of under-resourced countries, should adhere. These well-defined criteria are summarized through the ‘ASSURED’ (Affordable, Sensitive, Specific, User-friendly, Robust, Equipment-free, Deliverable) acronym [4]. The objective therefore for all developers has been to develop POC tests that are not only reasonably affordable but are also sensitive and specific enough for adoption in routine diagnostics.

Significant effort has therefore been made to develop such biosensors and one such example is paper-based microfluidic devices, which promise to satisfy these requirements. Their inherent advantages such as low-cost, ease of use, portability, requiring small sample volumes and no additional need for laboratory equipment and trained personnel have attracted attention within the diagnostics research domain as a low-cost alternative to conventional POC diagnostic tools [5,6,7].

Rapid Diagnostic Test (RDT) strips, also known as lateral flow devices (LFDs) are one of the simplest and most established formats of paper-based devices that allow the detection of an analyte through the testing of a complex bodily fluid such as urine, blood, saliva or even sweat [8,9,10,11]. The underlying principle of an LFD is relatively simple: a liquid sample containing the analyte of interest is moved via the paper-enabled capillary action through various zones within a paper strip. During the transport, the analyte interacts with antibodies that have been pre-deposited onto the strip and consequently gets captured at the detection sites namely the test line and the control line. The read-out is normally displayed within 5–30 min, and this is an indication of the detection of the analyte and manifests itself as an appropriate colour-based response at the test line, while a colorimetric response at the control line indicates that sample flow has correctly occurred through the strip and that the device has worked correctly [12]. Low development costs and ease of production of these LFDs have resulted in the expansion of applications across multiple test-sites where rapid tests are required, such as hospitals, physician’s offices, clinical laboratories and even in the patient’s home [13]. These tests are therefore now widely used as routine tools at the POC as part of an early-stage detection/treatment protocol, a common example of which is a pregnancy dip-stick [14].

Multiplexing, which is defined as simultaneous analysis of multiple analytes under the same set of conditions, is a critical parameter for increasing diagnostic efficiency [15]. An obvious example of such a need is in clinical diagnosis, where multiple analytes which are inter-dependent need to be detected and quantified to allow informed decisions to be made concerning the progression or stage of a particular disease [16]. In recent years, therefore, there has been an increasing demand for POC multiplexed diagnostic assays. The strategies that enable such simultaneous analysis of multiple analytes are largely based on the use of the following underlying diagnostic techniques, i.e., 96-well microtiter plate-based enzyme linked immunosorbent assays (ELISA) [17], real-time polymerase chain reactions (PCR) [18,19], and microarrays or bead-based methods [20,21]. Although all these methods could enable high-throughput and low volume processing, they require expensive non-portable equipment and trained personnel for their operation. Consequently, LFDs are regarded as one of the most competitive candidates for POC settings, due to their unique advantages that have been described earlier.

Currently, LFDs that provide such multiplexing are constructed either by laminating or shaping together different individual LFDs [22,23] or alternatively, by multiplexing within a single flow-path [24,25]. Such devices have inherent drawbacks such as, for the former, increased device dimensions and therefore need for larger sample volumes, and, for the latter, an undesired interference between different detection sites, i.e., the influence of each of the previous test-lines on subsequent lines positioned further along the flow-path. As a result, few commercial devices employing such multiplexing methodologies are available so far.

In this article, we propose a novel solution to overcome both of these limitations—a multi-path LFD created via the precise partitioning of the flow-path of a single LFD using a laser direct-write (LDW) technique. The multiple flow-paths allow individual detection of different analytes in each of the separated channels. We successfully demonstrate the use of these LDW-fabricated multi-path LFDs for simultaneous detection of a biomarker panel comprising C-reactive protein (CRP) and Serum amyloid A-1 (SAA1), commonly used for the diagnosis of bacterial infections. Unlike other multiplexing methods, our approach that is presented here, allows the creation of multiple individual flow-paths inside a ‘single’ LFD without increasing its original size. These multiplexed tests do not require multiple inlets and an increased sample volume, and, most importantly, eliminate the interference between individual detection sites positioned within the same channel. Overall, we believe that our LDW approach provides a novel and easy-implementable solution for enabling multiplexed detection via LFDs that hugely improves the detection efficiency and will therefore lead to an increase in the current market-size of LFD-based testing within in-vitro diagnostics.

## 2. Materials and Methods

### 2.1. Device Fabrication Setup

**Figure 1:**
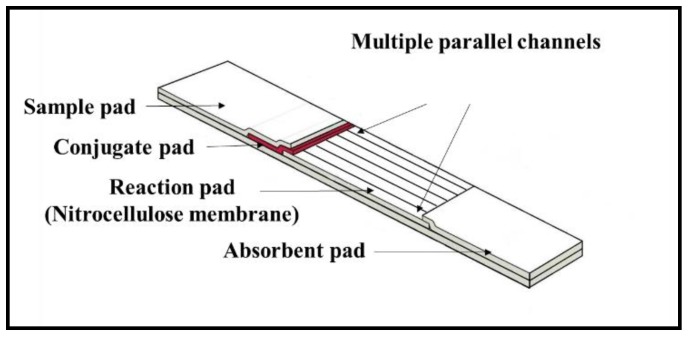
Schematic of a multiple (six) flow-path lateral flow device (LFD).

As shown in the schematic in Figure 1, the multi-flow path LFD was fabricated via precise partitioning of the nitrocellulose membrane (i.e., the reaction pad) of the single flow channel of a standard LFD into multiple separated flow-paths. As with the standard LFD, the sample dispensed onto the sample pad will flow further via capillary action into the conjugate pad where it picks up the pre-deposited reagent and then splits to flow into individual separated flow-paths created within the reaction pad allowing multiplexed detection without any interference or cross-reactions.

**Figure 2:**
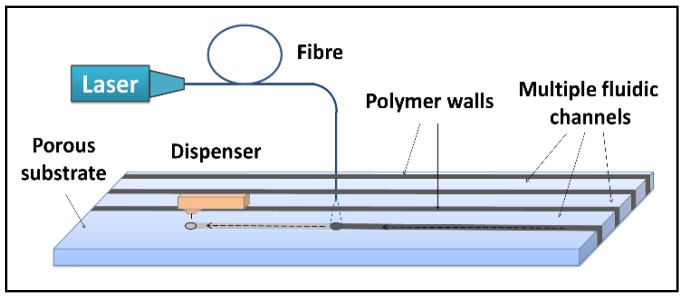
Schematic of the laser-based direct-write setup which shows the local deposition of photo-polymer that is subsequently illuminated by exposure from a 405 nm c.w. laser.

The boundaries that separate these individual channels were created using our previously reported local-deposition-assisted LDW technique [26,27,28]. The schematic of this local-deposition-assisted LDW setup is shown in Figure 2. A photo-polymer was first locally deposited onto the nitrocellulose reaction pad with a deposition nozzle at locations pre-defined by the device design. A laser beam that follows the deposition head subsequently illuminated the deposited polymer pattern inducing photo-polymerisation. These laser-cured patterns that extend through the thickness of the nitrocellulose membrane define the solid walls of the multiple fluidic channels that confine and transport individual liquid flows.

The laser used for this LDW process was a 405 nm continuous wave (c.w.) diode laser (MLDTM 405 nm, Cobolt AB, Stockholm, Sweden) with a maximum output power of 110 mW). The photopolymer used for creating the boundary walls between individual channels was DeSolite*^®^* 3471-3-14 from DSM Desotech, Inc., Elgin, IL, USA. The dispenser platform used for the local deposition of the photopolymer onto the various substrates was a PICO*^®^* Pµlse™ dispensing system from Nordson EFD, UK. A different reagent-dispensing system was used for local deposition of antibodies onto the reaction pad for creation of test lines and control line and that was the XYZ3210 dispense platform from Biodot, Irvine, CA, USA.

### 2.2. Reagents and Materials

The nitrocellulose membrane used as the reaction pad was UniSart CN95 purchased from Sartorius and the absorbent pad was cellulose filter papers (CF1) from GE Healthcare. For the CRP assay, the capture and detection antibodies used were mouse anti-human CRP antibody (MAB17071) and biotinylated mouse anti-human CRP antibody (BAM17072) from R&D Systems, Abingdon, UK. The protein standard was recombinant human CRP expressed in *E. coli* (Sigma Aldrich C1617). For the SAA1 assay, both the capture and detection antibodies and also the protein standard used were from a DuoSet ELISA kit (DY3019) from R&D Systems. The control line antibody was anti-mouse Goat IgG (AF007) from R&D Systems. The streptavidin-conjugated gold nanoparticles (with an optical density of 10) were obtained from BBI solutions (BA.STP40). The diluent used for preparing all the antibody and sample solutions was 1% BSA in PBS. The bovine serum albumin (BSA) (A2058) and phosphate buffered saline (PBS) (P3831) used were obtained from Sigma Aldrich, Gillingham, UK.

## 3. Results and Discussion

**Figure 3:**
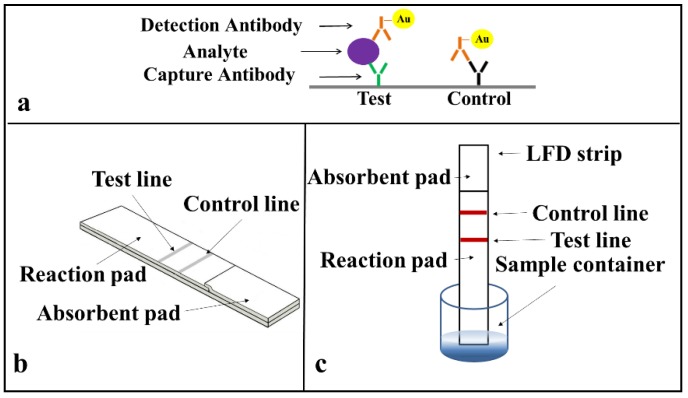
Schematic of (**a**) the sandwich enzyme linked immunosorbent assays (ELISA) protocol, (**b**) the simplified LFD we used for detection of C-reactive protein (CRP) and Serum amyloid A-1 (SAA1), and (**c**) detection operation method.

Prior to developing the multiplexed LFD, we first demonstrated the successful implementation of single assays on a standard LFD using a sandwich ELISA for the two identified biomarkers (CRP and SAA1) commonly used as indicators of an inflammation. The protocol for the sandwich-ELISA we have used is described in Figure 3a. The target analyte (CRP or SAA1 protein) is sandwiched due to antigen–antibody binding between the capture and the detection antibody a, which is pre-tagged with an Au-nanoparticle via a biotin-streptavidin binding. This binding and the resultant accumulation of Au-nanoparticles at the test line produce the appearance of a red colour at these lines.

The arrangement of a simplified standard lateral-flow strip that we used for these single detections is as shown in Figure 3b and only consists of a reaction pad made of nitrocellulose membrane and an absorbent pad that is made of cellulose paper. For these proof-of-principle test to ease with the assay procedure, we simplified the design of the LFD by eliminating the sample pad and conjugate pad. These single LFDs have a dimension of 3 mm in width and 5 cm in length, which are chosen according to the standard commercially available LFDs in market. The capture antibody was pre-deposited and immobilised at the test line and a mixture of the sample and the Au-nanoparticle tagged detection antibody was introduced directly via the reaction pad.

The operation of this LFD is described in Figure 3c. When the strip is dipped into a mixture of the sample containing the analyte and the Au-nanoparticle tagged detection antibodies, an analyte-antibody-Au nanoparticle complex is formed via the specific chemical binding between the analyte and the tagged-antibody. This complex then flows laterally via capillary action towards the absorbent pad that is positioned at the other end of the lateral-flow strip. As the complex migrates through the reaction pad, the analyte binds to the capture antibody that is pre-immobilized in the reaction zone and the excess sample-reagent mixture then wicks further and finally gets collected by the absorbent pad. The accumulation of the Au-nanoparticle tagged antibody-analyte complex produces the appearance of a red colour test line that indicates the presence of the target analyte in the sample.

### 3.1. Standard Single LFD for Detection of CRP

We first studied the detection of CRP using single LFDs. The capture antibody (mouse anti-human CRP at 1 mg/mL) was locally deposited onto the reaction pad as a line using the BioDot dispenser and left to dry and immobilise overnight. A mixture of the sample (containing the analyte, a recombinant human CRP protein in various concentrations ranging from 10 μg/mL to 50 ng/mL), the detection antibody (biotinylated mouse anti-human CRP at 1.6 μg/mL), and the streptavidin-modified 40 nm Au-nanoparticles in a volumetric ratio of 3:2:4 (values which we had optimized through previous experiments) was first prepared immediately prior to the testing with the single LFDs.

**Figure 4:**
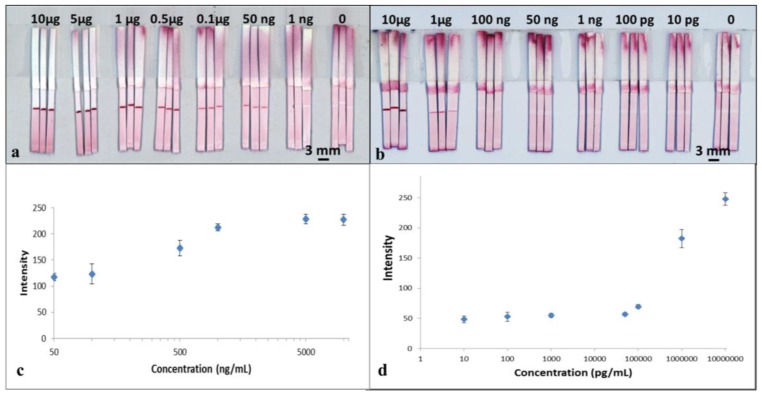
Scanned images of sets of the standard single (**a**) CRP and (**b**) SAA1 LFDs with various CRP and SAA1 analyte concentrations, in each case per ml. (**c**,**d**) show calibration curve constructed using the grayscale intensity values taken from the image shown in (**a**,**b**). Error bars indicate the standard deviation for 3 individual measurements.

To perform the test, 15 μL of the mixture was added into a well of a 96-well microtiter plate and then the LFD was dipped into the sample solution. The device was left to run for 3 min to allow all of the solution in the well to be wicked into the strip. The device was subsequently dipped in a chase buffer of PBS solution for another 5 min to allow the entire reagent to be pushed through to the reaction pad. The result was then immediately captured using a scanner (Epson Perfection V800 Photo A4 Flatbed Scanner). The resultant, appearance of the red-coloured test lines are as shown in Figure 4a. For samples with concentration of 50 ng/mL, the red-coloured test lines however appear within a larger white band around them. The formation of the white bands is a consequence of the blocking of that area/band around the test line by the BSA contained within the capture antibody solution previously dispensed to produce the test line. BSA is a reagent most commonly used to block unwanted nonspecific binding sites on the nitrocellulose membrane 29. A similar white band, though smaller in size, is also visible for devices tested for higher analyte concentrations.

The images captured using the scanner were processed with the ImageJ software (National Institutes of Health, Bethesda, MD, USA) to extract the respective colour intensities of the red colour produced at the test lines. The resultant plot in Figure 4c shows the relationship between the respective test-line colour intensities for each of the individual devices and the corresponding concentrations for the samples used to test each of the devices.

The results above show the successful detection of CRP using a standard single flow-path LFD. From the plot in Figure 4c, we observe that the intensity of the test line shows a monotonic increase as a function of concentration of the sample, which shows its potential usefulness in quantitative analysis. With these standard single-channel LFDs, we have shown the capability for detection of CRP with a concentration down to 50 ng/mL, which is comparable with commercial ELISA assays performed on microtiter plates.

### 3.2. Standard Single LFD for Detection of SAA1

Following the experiments for the CRP detection using standard single-channel LFDs, in a similar manner, we then tested their use for detection of SAA1. These LFDs had the same device geometry as those used previously for the CRP testing. The capture antibody used was mouse anti-human SAA1 at 1 mg/mL. The sample mixture was prepared by mixing of the sample (containing the analyte, a recombinant human SAA1 protein with various concentrations ranging from 10 μg/mL to 10 pg/mL), the detection antibody (biotinylated mouse anti-human SAA1 at 12 μg/mL) and the Au-nanoparticles (streptavidin-modified 40 nm gold nanoparticles) in a volumetric ratio of 3:2:4.

The tests were performed in the same way as described for CRP testing by dipping the LFDs into the sample-containing well followed by dipping in a chase buffer. The results are shown in the scanned image in Figure 4b. The image was processed using ImageJ and the data is plotted in Figure 4d. As seen in Figure 4b, for samples with concentrations below 1µg/mL, once again the red-coloured test lines appear within a larger white band.

From both the images in Figure 4b and the plot in Figure 4d, we can see that although the test line does appear for concentrations of 50,000, 1000, 100 and 10 pg/mL, the colour intensity level is low and fairly constant, which thereby limits any quantitative analysis. However, our results show the capability of a qualitative (YES/NO type) detection of SAA1 for concentrations as low as 10 pg/mL, which is more than three orders of magnitudes lower than what can be measured using commercially available ELISA kits.

The clinically relevant ranges for CRP and SAA1 are 50 ng/mL to 500 µg/mL [29] and 1 µg/mL to 5 mg/mL [30] respectively, and the results above show that while our standard single LFDs cover a section of the relevant range they also span across the comparatively challenging lower concentration range indicating their lower limit-of-detection. We chose not to work across the high concentration ranges as such levels are only observed in patients having acute infection and such concentrations can be detected simply via pre-dilution of the sample, which is a routinely applied procedure when the concentration of antigen in the sample potentially exceeds the highest point of the standard curve [31].

### 3.3. Multiplexed Detection of CRP and SAA1 in Single-Channel LFDs

After demonstrating the successful detection of the above two analytes in separate LFDs, we then attempted their combined detection using a single-channel multiplexed LFD via the introduction of multiple detection lines within the common reaction zone of a standard single LFD.

**Figure 5:**
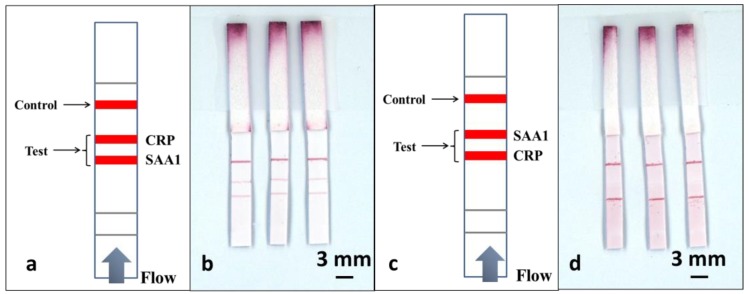
Schematic of a single-channel multiplexed LFD with multiple test lines along the same flow-path for multiplexed detection of CRP and SAA1, (**a**) with the SAA1 test line being before that for CRP and (**c**) with the CRP test line before that for SAA1. (**b**,**d**) are scanned image shows the results for three such LFDs described in (**a**,**c**).

As shown in Figure 5a, to perform this multiplexed detection, we used a multiple test line format. Along the direction of the flow, the test line for SAA1 was positioned 3mm before the test line for CRP, and finally, a control line was positioned 4 mm after the CRP test line. The sample was a pre-prepared mixture of CRP and SAA1 in a 1:1 ratio, with analyte concentrations of 100 ng/mL and 1 µg/mL respectively.

Based on the results from our previous section, for these experiments we chose to test for concentrations of 100 ng/mL and 1 µg/mL for CRP and SAA1 respectively, which as shown in Figure 4a,b, have relatively comparable colour intensity levels.

To perform the tests, we have use an identical volume of 15 µL for the sample-reagent mixture and the results are shown in the scanned image in Figure 5b. ImageJ was used to extract the colour intensity of both the test lines corresponding to CRP and SAA1and then these values were compared with the values previously obtained from the calibration curves in Figure 4c,d. We found that the intensity of the SAA1 test line that is located before the CRP test line stays at a level similar to what was achieved using a standard single-path SAA1-LFD, while the intensity of the CRP test line dropped significantly (more than threefold) when compared with the result for the standard single-path CRP-LFD.

Based on these results we can infer that the second test line is affected by the presence of a first test line positioned prior to it in the flow-path. To further confirm this hypothesis, we then used the same devices and reversed the position of the two test lines, as shown in Figure 5c, where the test line for CRP precedes the SAA1 test line. The devices were tested by dipping into the same 15 µL of sample-reagent mixture used for the previous experiments and the result is shown in Figure 5d. When compared with the image shown in Figure 5b, we can clearly observe that the test line for the SAA1 is almost invisible, whereas the intensity of the CRP test line positioned prior to the SAA1 test line shows a significant enhancement. Again by extracting the colour intensity using ImageJ, we found that the CRP test line has the same level of intensity as for the standard single LFD and the intensity for the SAA1 test line dropped by almost 7 times.

These results clearly confirmed here the problem of undesired interference between sequential detection sites positioned within a common flow-path, namely, the influence of a test-line on subsequent lines positioned further along the flow-path. This represents a key barrier to the use of such a single-channel LFD with sequentially positioned test lines as an appropriate tool for multiplexed detection. An alternative, as proposed by others to achieve multiplexed LFDs is to laminate or shape together different individual LFDs. However, this leads to an increased device footprint and therefore requires larger sample volumes, which is also undesirable, especially when working with samples such as blood or saliva etc. that normally are available in limited volumes.

### 3.4. Integrated Dual-Channel LFDs for Multiplexed Detection of CRP and SAA1

To solve this problem of interference, we propose herein the use of our LDW method for creation of multiple flow-paths in a standard single LFD that allows isolation of each of those different detection sites, without increasing the overall dimension of the original device.

**Figure 6:**
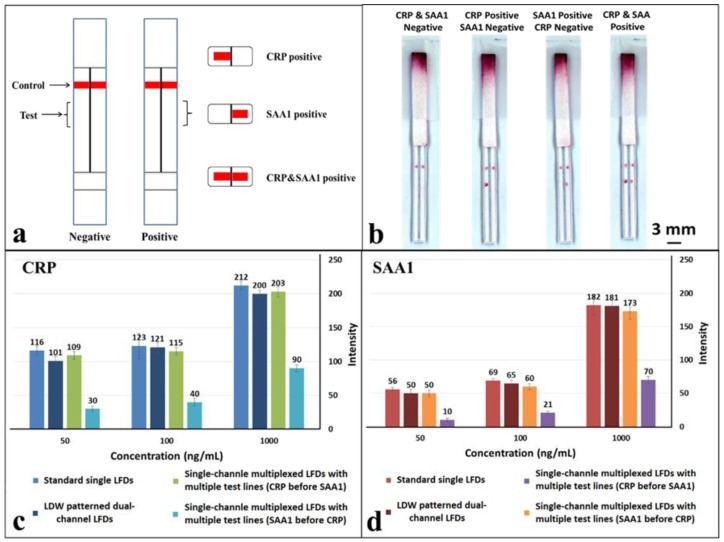
(**a**) Schematic of the laser direct-write (LDW) patterned multiplexed LFD with multiple channels for multiplexed detection of CRP and SAA1; (**b**) scanned image shows the multiplexed detection of CRP and SAA1 using LDW patterned dual-channel LFDs described in (**a**); (**c**,**d**) plots showing direct comparison of the measured colour intensities of test lines that appeared in standard single LFDs, single-channel multiplexed LFDs and our LDW-patterned dual-channel LFDs for detection of CRP & SAA1 respectively at concentrations of 50 ng/mL, 100 ng/mL and 1 µg/mL. Error bars indicate the standard deviation for 3 individual measurements.

As shown in the schematic in Figure 6a, a hydrophobic photopolymer barrier was patterned along the middle of the (reaction pad) nitrocellulose membrane to partition the original single flow channel into two parallel isolated flow-paths where individual test lines can be incorporated for detection within these separated channels. Here, we use this device with two flow-paths as an example to implement multiplexed detection of CRP and SAA1. The capture antibodies for both test lines were locally deposited into individual channels, as shown in the schematic with the left channel for CRP and the right for SAA1. The appearance or absence of either test line signifies the presence or absence of either marker in the sample.

The tests were performed in the same way as for the previous multiplexed test single-channel LFD by dipping the strip into a 15 µL sample-reagent mixture and the results are shown in Figure 6b. Based on the simultaneous appearance/absence of test lines in these separated channels, we can then identify the sample to be either CRP/SAA1 positive or positive/negative for both. As can be seen in the image (Figure 6b), both the test lines, for CRP in the left channel and SAA1 in the right channel have almost equal intensities to those for corresponding test lines within the standard single LFDs (Figure 4a,b) that we have previously tested.

To further confirm the above observations, the colour intensities of the test lines in these LDW-patterned dual-channel LFDs were measured and then compared with those acquired for both the standard single LFDs and single-channel multiplexed LFDs. The three concentrations used for these comparisons were 50 ng/mL, 100 ng/mL and 1 µg/mL for both CRP and SAA1. These results are shown in the plots in Figure 6c,d. For CRP (Figure 6c), at the same concentration, the intensity of the test line in a single-channel multiplexed LFD with the SAA1 test line before that for CRP dropped significantly to less than half of that for a standard single LFD. The colour intensity of the test line for both the single-channel multiplexed LFD with CRP test line before that for SAA1 and our LDW patterned dual-channel LFD are at the same level as for the standard single LFD. Furthermore, the trend for the colour intensity levels for SAA1 (Figure 6d) is also the same as that observed for CRP.

Overall, as anticipated, these results conclusively confirm that there is no interference between two test lines placed within the individual channels of the dual-channel LFD, because both detections are implemented independently in parallel channels rather than in series within a single channel. Additionally, by patterning more than a single barrier in the reaction pad more flow-paths can be created hence allowing multiplexing of more than this proof-of-principle dual assay. In the current dual-channel LFDs, the laser-patterned barrier walls have dimensions of ~500 µm, however, this can be reduced to have dimensions of ~200 µm if desired. It is therefore then possible to pattern a greater number of fluidic flow-paths while maintaining the widths of our LFDs to be similar to that of a standard single LFD (that has a width of ~5mm)―allowing detection of not two, but a greater number of analytes from within the same sample.

## 4. Conclusions

In conclusion, we have proposed a novel solution to achieve multiplexed diagnosis in LFDs that overcomes the limitations of current techniques including either interference of multiple test sites positioned in the same flow-path or increased device dimensions that require larger sample volumes. We have developed a LDW technique that allows creation of multiple flow-paths in a single LFD simply via the precise partition of the flow-path of a single LFD. The multiple isolated parallel flow-paths then allow individual detection of the different analytes in each of the separated channels without interference or cross-reaction. As a proof of principle, we have shown the successful use of a dual-channel LFD for multiplexed detection of a biomarker panel comprising CRP and SAA1, used commonly for the diagnosis of bacterial infections. The results show that our laser-patterned LFDs performed equally well as the single LFDs and do not need increased device dimensions or additional sample volumes. Overall, we believe that when compared to the current multiplexed detection procedures our technique offers a better solution for multiplexed detection within LFDs.
